# Digestibility and Quality Characteristics of Noodles with Added Malic-Acid-Modified Wheat Starch

**DOI:** 10.3390/foods14081348

**Published:** 2025-04-14

**Authors:** Gyeong A Jeong, Inae Lee, Chang Joo Lee

**Affiliations:** 1 Department of Food Science and Biotechnology, Wonkwang University, Iksan 54538, Jeonbuk, Republic of Korea; jka0719@naver.com; 2 Department of Food Science and Biotechnology, Kyung Hee University, Yongin 17104, Gyeonggi-do, Republic of Korea; ilee3873@khu.ac.kr

**Keywords:** resistant starch, noodle, wheat starch, malic acid, thermostable

## Abstract

Starch digestion raises blood glucose levels and is associated with cardiovascular diseases, diabetes, metabolic syndrome, and obesity. Hence, developing methods for controlling starch digestion is important. In this study, we prepared noodles from wheat flour containing malic-acid-modified starch (MAS), which contains a high proportion of thermostable resistant starch (RS). The quality and digestibility characteristics of these MAS-added noodles were evaluated to determine the optimal formulation. MAS was prepared by physicochemically modifying native wheat starch by adding 4 M malic acid and heating at 130 °C for 7 h. MAS-added noodles were produced by preparing a flour mixture in which 5–30% wheat flour was replaced with MAS. Compared to traditional wheat flour noodles, the addition of MAS resulted in inferior texture, extensibility, and cooking properties, along with higher solid losses, which negatively affect noodle quality. Nevertheless, less-rapidly digestible starch and more RS were observed at higher MAS levels. The inclusion of 10% MAS was found to afford the highest RS content while maintaining noodle quality similar to that of commercially available wheat flour; this formulation was determined to be optimal for producing MAS-added noodles. Therefore, MAS, with its enhanced thermostable RS content, is a promising low-calorie ingredient for use in the food industry. Further research into MAS and the development of MAS-based food products may promote the development of new and diverse low-calorie food options.

## 1. Introduction

Starch is a type of carbohydrate composed of glucose-based polysaccharides, specifically amylose, which consists of glucose units linked by α-1,4 glucosidic bonds, and amylopectin, which is linked by both α-1,4 and α-1,6 glucosidic bonds [1,2]. Starch is a primary energy source for the human body and is found in various plants, such as rice, wheat, corn, and barley [3]. The nutritional properties of carbohydrates depend on their digestion and absorption rates, and disaccharides and polysaccharides must be broken down into monosaccharides for absorption [4]. The digestibility of starch is influenced by its granular structure, amylose content, and amylopectin composition [5]. Starch digestion is categorized into three types based on the rate and extent of digestion: rapidly digestible starch (RDS), which is digested within 20 min of ingestion; slowly digestible starch (SDS), which is digested for 20–120 min; and resistant starch (RS), which remains undigested beyond 120 min and is fermented in the colon [6]. RDS is rapidly digested and absorbed in the duodenum and proximal small intestine, leading to a sharp increase in blood glucose levels [7], which are associated with cardiovascular diseases, diabetes, metabolic syndrome, and obesity [4,8]. In contrast, SDS and RS help regulate postprandial blood glucose levels; consequently, they are suitable for patients with diabetes and as low-calorie dietary components of weight-management products [9,10]. RS is resistant to enzymatic digestion in the human gastrointestinal tract, resulting in slow glucose release and incomplete degradation in the small intestine. Instead, RS is fermented by gut microbiota in the colon to produce short-chain fatty acids that promote the growth of beneficial bacteria [11]. This process contributes to the prevention of cancer, improved blood glucose regulation, and weight management in obese individuals, owing to its low-calorie properties [10]. Starch-containing foods are generally consumed in cooked form, during which starch undergoes gelatinization and in which SDS and RS are converted into RDS. This transformation results in a loss of the functional benefits related to digestion and absorption in the human body [8]. Consequently, a heat-stable resistant starch capable of regulating postprandial blood glucose levels even after cooking (or gelatinization), has been developed to address this issue by modifying starch [12,13,14,15,16].

Modified starch is produced through chemical, physical, or enzymatic modifications, and a combination of these methods is used to enhance its resistance to digestion [17]. Chemically modified starch is primarily produced using chemical or organic acids because they lead to less starch hydrolysis [18,19]. Acid-treated modified starch exhibits an enhanced RS content, thereby preserving the health benefits of the RS [11,20]. Research into RS-enriched modified starch has focused on its use in various food products, such as cookies, yogurt, cakes, and noodles, with the aim of maximizing its functional health benefits [21,22,23,24]. Chae et al. [25] investigated the quality characteristics of cookies containing octenyl succinyl anhydride-modified wheat starch, while Alexander et al. [26] evaluated the noodle-making potential and digestibility of noodles prepared with 15–30% citrate-modified oat starch. Malic acid, a food-grade C4-dicarboxylic organic acid, has also gained popularity for acid modification of starch [27]. Recent studies have optimized modification conditions—including pH, concentration, treatment temperature, and time—to maximize resistant starch content, further demonstrating its high potential as a functional food ingredient [28,29].

Noodles are a staple food in many Asian countries because they are convenient, easy to cook, and affordable [24]. Noodles primarily consist of carbohydrates; therefore, partially substituting their ingredients with functional carbohydrates, such as RS, is unlikely to result in consumer resistance. Moreover, RS-enriched noodles suppress postprandial blood glucose spikes and contribute to a low-calorie diet. Although previous studies have examined the physicochemical properties, digestibility, and thermal stability of modified starch treated with malic acid and heat [9,29], limited research into its use in food products and its potential as a functional food ingredient has been reported. Therefore, in this study, we developed malic-acid-modified starch (MAS) that contains up to 99.5% RS with thermostable properties, which was prepared under the optimal malic-acid-modification conditions identified in our previous study [29]. MAS-added noodles were formulated by replacing 5–30% of its wheat flour with MAS. The quality characteristics of MAS-added noodles were evaluated to determine the optimal substitution level that balances desirable noodle properties with the functional benefits of resistant starch.

## 2. Materials and Methods

### 2.1. Experimental Materials

Common wheat flour (CJ Cheiljedang, Yangsan, Republic of Korea) was used for starch-isolation and noodle-preparation purposes. dl-Malic acid (M1210, Sigma–Aldrich, St. Louis, MO, USA) was used to produce RS-enhanced modified wheat starch. Porcine pancreatin (P7545; activity, 8 × United States Pharmacopeia [USP]/g; Sigma–Aldrich, St. Louis, MO, USA) and amyloglucosidase (AMG 300 L; activity, 300 amyloglucosidase activity [AGU]/mL; Novozymes Inc., Bagsvaerd, Denmark) were used in starch-digestibility experiments.

### 2.2. Isolating Starch

Wheat starch was isolated using the double-washing method described by Kim and Huber [30] with some modification. Specifically, flour (1 kg) was mixed with distilled water (500 g) and kneaded for 20 min to facilitate the formation of gluten. The dough was then placed in a stainless-steel bowl filled with water, and the starch and gluten were separated using a 150-mesh sieve. The starch suspension was centrifuged (Supra R22; Hanil Scientific Inc., Gimpo, Republic of Korea) at 5000× *g* for 15 min, and the supernatant was removed. The yellow protein and tailed-starch layers on the top of the sediment were scraped off with a spatula. The starch was then resuspended in distilled water and repeatedly washed until no protein or tailed-starch layer was observed. The isolated starch was completely dried at 45 °C and then passed through a standard 150-mesh sieve (No. 150; Chunggye, Seoul, Republic of Korea) before use in any experiment.

### 2.3. Modifying Starch with Malic Acid

MAS was prepared by physicochemically modifying starch under the optimal conditions described by Mansur et al. [29], which produced the highest RS content. Starch (20 g) and 4 M malic acid (20 mL) were placed in a stainless-steel dish, the pH was adjusted to 1.2 using 10 M NaOH, and the mixture was soaked at room temperature for 16 h, after which it was dried in a hot-air oven (C-DF3, Changshin Science, Seoul, Republic of Korea) at 45 °C until the moisture content was below 10%. The residue was finely ground in a blender and allowed to react at 130 °C for 7 h using the hot-air oven. The malic acid was removed by washing with distilled water and rinsing with 95% ethanol. The malic-acid and thermally treated starch was then dried in a hot-air oven at 45 °C until the moisture content was below 10%. The resultant dried starch block was ground and passed through a 150-mesh sieve to produce MAS powder for use in subsequent experiments. The RS content of the MAS was determined to be 99.5% through starch digestibility analysis, which is consistent with the findings of Mansur et al. [29].

### 2.4. Preparation of MAS-Added Noodles

Wheat-flour–MAS mixtures were prepared by partially substituting wheat flour with various amounts of the prepared MAS: 5% (MAS-5), 10% (MAS-10), 20% (MAS-20), and 30% (MAS-30) (Table 1). Doughs were formed by mixing salted water and various wheat-flour–MAS mixtures for 5 min at room temperature using a noodle mixer (KMM020, Kenwood, UK). Each dough was shed using a noodle machine (HSN-2; Hunwoo, Seoul, Republic of Korea) with sequential roller gaps of 7.5, 5.0, 4.0, 3.3, and 2.7 mm. The dough was sliced into fresh 3-mm-wide, 2.2-mm-thick noodles. For comparison, wheat noodles devoid of starch (Wheat) and control noodles containing 20% native starch (Control) were used as references. To ensure the reproducibility and reliability of the experimental results, both the MAS-added noodles and the control noodles were repeatedly produced using the same flour and ingredients on the same date and production line.

### 2.5. Color and Appearance of the MAS-Added Noodles

The color of each 2.2-mm-thick noodle sheet was measured using a colorimeter (Model CM-5, Minolta Co., Tokyo, Japan) before and after being cooked. The colorimeter was calibrated using a standard white plate, and Hunter values (L* (lightness), a* (redness), and b* (yellowness)) were recorded. The total color difference (Δ*E*) was calculated using the following equation:

(1) $$\Delta E=\sqrt{L^{2}+a^{2}+b^{2}}$$

### 2.6. Texture Profiles of the Cooked Noodles

The textures of the cooked noodles were measured using a texture analyzer (TA-XT2, Stable Micro Systems, Godalming, Surrey, UK) in texture-profile-analysis (TPA) mode. Noodle cooking time was defined as the time at which the internal and external regions of the noodles exhibited identical colors. Consequently, the noodles were cooked in boiling purified water (100 °C) for 12 min, rinsed with cold water for 1 min, and drained for 3 min prior to analysis. Measurements were performed three times per sample using a cylindrical probe (P/35, 35 mm diameter). Four noodle strands, each 5 cm long, were placed in parallel on a plate. The noodles were then subjected to two compression cycles until their surfaces were deformed by 70% of their total thickness. The hardness, springiness, cohesiveness, gumminess, and chewiness of the noodles were recorded according to the conditions listed in Table 2.

### 2.7. Tensile Strengths of the Cooked Noodles

Tensile strength was measured using a Texture Analyzer™ (TA-XT2) equipped with a noodle tensile rig. Each measurement was repeated three times. Noodles were cooked under the same conditions as described above, after which a single noodle strand was placed between clamps with a 20-mm gap, and the force (N) required to break the noodle and the elongation distance (mm) were recorded.

### 2.8. Cooking Properties of the Noodles

Cooking properties were evaluated in terms of differences in weight, volume, and water-absorption of the cooked noodles according to the method of Kim et al. [31], with some slight modifications. Uncooked noodles (25 g) were boiled in purified water (500 mL) for 12 min, rinsed with cold water for 1 min, and drained for 3 min, after which the cooked noodles were weighed. The noodle volume was measured by immersing the cooked noodles in purified water (150 mL) in a 250-mL graduated cylinder and recording the volume increase. Water absorption was calculated using the following formula:

(2) Water absorption%=Weight of cooked noodles−Weight of uncooked noodlesWeight of uncooked noodles×100

### 2.9. Amount of Leached Solids

The amount of leached solids was determined according to the method of Kim et al. [31], with some slight modifications. Uncooked noodles (25 g) were boiled in purified water (500 mL) for 12 min, after which the volume of the cooking water was adjusted to 500 mL with purified water. The turbidity of the cooking water was measured at 675 nm using a spectrophotometer (UV-1080, Shimadzu Co., Kyoto, Japan).

### 2.10. In Vitro Noodle Digestibility

The RDS, SDS, and RS contents of the cooked MAS-added noodles were determined following the methods of Englyst et al. [32] and Shin et al. [33], with some modifications. Freeze-dried noodle samples were ground and passed through a 150-mesh sieve. Starch digestibility was measured using a pancreatin-based enzyme solution. Sodium acetate buffer (0.75 mL, 0.1M, pH 5.2) and the pancreatin-based enzyme solutions (0.75 mL) were added to the freeze-dried noodle sample (30 mg) in a 2-mL tube along with type-4 glass beads. The reaction was carried out for 20–240 min in a shaking incubator (VS-8480SF; Vision Scientific Co., Bucheon, Republic of Korea). Following the reaction, enzyme activity was stopped by heating the mixture at 110 °C in a heating block for 10 min and then cooling it to room temperature. The mixture was centrifuged and the glucose content in the supernatant was measured at 505 nm using a UV-Visible spectrophotometer (UV-1800, Shimadzu Co., Kyoto, Japan) following processing with a glucose-oxidase–peroxidase (GOD–POD) kit (Embiel Co., Gunpo, Republic of Korea). The enzyme-resistant starch content was calculated using the program provided in the GOD–POD kit (Embiel Co., Gunpo, Republic of Korea). The amount of glucose obtained after 20 min of enzyme reaction at 37 °C corresponds to the RDS, and that obtained after incubation for 20–240 min corresponds to the SDS. The RS had not been hydrolyzed after 240 min of incubation.

### 2.11. Statistical Analysis

All experimental results are expressed as the means ± standard deviations (SDs) of three replicates. Statistical significance was determined using one-way ANOVA followed by a Duncan’s multiple range test using SPSS (version 23.0; SPSS Inc., Chicago, IL, USA). Differences were considered statistically significant at *p* < 0.05.

## 3. Results and Discussion

### 3.1. Color and Appearance Characteristics of the MAS-Added Noodles

The color characteristics of MAS-added noodles are presented in Table 3. Colors are classified in terms of lightness (L*), redness-greenness (a*), and yellowness-blueness (b*). Noodles containing chemically modified starch have been reported to become increasingly white in color with increasing levels of modified starch [34]. In a similar manner, we observed lightness values that increased in moving from uncooked wheat (76.5) to MAS-5 (82.1), MAS-10 (85.4), MAS-20 (86.5), and MAS-30 (88.2) noodles; this increase in whiteness is likely ascribable to the inherent whiteness of starch [34]. The redness and yellowness of uncooked noodles tended to decrease as the MAS content increased. This result is consistent with the previous findings that the addition of starch to noodles leads to a reduction in redness and yellowness [35]. Cooked noodles are less light, red, and yellow overall than uncooked noodles, owing to moisture absorption, leaching, and cooking characteristics [36,37]. Noodle color depends on the additive type and concentration [38,39]. Therefore, the color changes in MAS-added noodles are primarily attributed to the inherent whiteness, water absorption, and leaching characteristics of the MAS starch, which likely contributed to an increase in brightness while simultaneously decreasing redness and yellowness.

**Figure 1:**
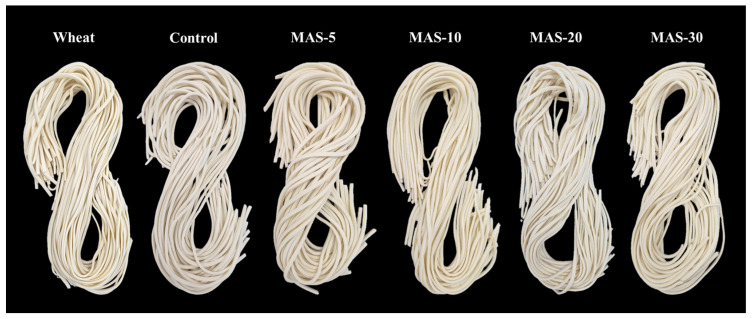
Appearances of noodles with various MAS contents. Wheat: 100% wheat flour; Control: wheat flour containing 20% native wheat starch; MAS-5–30: wheat flour containing 5–30% MAS.

Photographic images of the prepared noodles are shown in Figure 1. While the Wheat and Control samples exhibit good noodle-strand formation, the strand-forming abilities of the MAS-added noodle samples were observed to decrease with increasing MAS content, leading to noodle breakage during cooking at 30% MAS loading (MAS-30). This observation is likely due to the lower viscosity and reduced water-absorbing capacity of chemically modified starch compared to those of native starch, resulting in weaker binding. Treatment with malic acid and heat partially disrupts the starch molecular structure, decreasing the intensity of X-ray diffraction peaks and crystallinity [29]. These structural changes have been shown to contribute to reductions in swelling power and viscosity [29]. Therefore, the MAS content needs to be maintained below 30% to ensure proper noodle-strand formation.

### 3.2. Textures of the MAS-Added Noodles

The textural characteristics of the prepared noodles are presented in Table 4, which reveals that hardness decreases in the order: Wheat (28.3 N), MAS-5 (28.2 N), MAS-10 (27.1 N), MAS-20 (17.0 N), Control (15.7 N), and MAS-30 (11.3 N). In a similar manner, springiness, cohesiveness, chewiness, and gumminess also decreased from those recorded for wheat with increasing MAS content. These findings are consistent with those of Hong et al. [40], who reported that noodles supplemented with starch exhibit lower hardness, springiness, cohesiveness, chewiness, and gumminess than noodles prepared solely using wheat flour. Typically, noodle formation relies on the cohesive and elastic properties of gluten, the main protein component of wheat, which enhances dough structure and elasticity [41]. In contrast, due to the lack of gluten in starch, many attempts have been made to improve the quality of starch-based products by imparting thermal, rheological, and physical properties [42]. In this study, we chemically modified MAS to increase its RS content. However, the lower viscosity-forming ability of chemically modified starch led to an overall decrease in textural quality [9]. Therefore, MAS-5 and MAS-10, whose properties do not significantly differ from those of wheat, were considered to contain appropriate supplementation levels.

### 3.3. Tensile Strengths of the MAS-Added Noodles

The tensile strengths of the prepared noodles are listed in Table 5; these values were evaluated through compression-mode testing, in which the force (N) required to break each noodle (which reflects its rheological properties) was measured [35]. The tensile strength of the wheat noodles was determined to be 0.379 N, whereas the supplemented noodles exhibited a decreasing trend with increasing MAS content: MAS-5 (0.149 N), MAS-10 (0.124 N), MAS-20 (0.063 N), and MAS-30 (not measurable). The tensile strength of the MAS-30 sample was unable to be measured owing to insufficient noodle formation (i.e., they broke) during cooking. Chemical modification enhances the physical, functional, and chemical properties of native starch. However, starch has been reported to degrade (acid hydrolysis) when modified under strongly acidic conditions, resulting in a lower swelling capacity and viscosity [29,42]. Therefore, MAS-5 and MAS-10 are the most suitable options for achieving a tensile strength and texture similar to those of wheat.

### 3.4. Cooking Characteristics of MAS-Added Noodles

The cooking properties of the MAS-added noodles and their amounts of leached solids are presented in Table 6. The weight, volume, and water absorption of the cooked wheat noodles were determined to be 54.7 g, 195 mL, and 118%, respectively. The MAS-added noodle samples exhibited increasingly lower values compared to those of the wheat sample with increasing MAS content. Some solid noodle components may be lost during the cooking process, and the leached amount can be assessed by measuring the turbidity of the water following noodle cooking [35,43]. Turbidity was observed to increase with increasing MAS content, as follows: Wheat (0.185), Control (0.194), MAS-5 (0.262), MAS-10 (0.422), MAS-20 (0.637), and MAS-30 (1.282), which reveals that the MAS-added noodles leach more solids. MAS becomes esterified and cross-linked through reactions involving malic acid and the hydroxyl groups (-OH) of starch [9,13]. A high degree of crosslinking within the MAS inhibits starch-granule swelling, which reduces viscosity and hinders the formation of a binding network within the noodle dough [29,44]. Consequently, water absorption decreases with increasing MAS content, whereas turbidity increases owing to a greater level of leaching.

### 3.5. Starch Digestibilities of the MAS-Added Noodles

The digestibilities of cooked MAS-added noodles are shown in Table 7. Native starches with high RS contents (types 1 and 2) are poorly thermally stable and are rapidly converted into RDS during heat treatment [8]. To address this issue, more thermally stable RSs have been produced through chemical, physical, and enzymatic modifications [12,17]. The cooked noodles were determined to have the following RS contents: Wheat (18.1%), Control (17.9%), MAS-5 (22.0%), MAS-10 (25.9%), MAS-20 (34.7%), and MAS-30 (43.3%). The MAS-added noodles were found to contain 3.9–25.2% more RS than wheat noodles, with MAS addition observed to contribute to higher RS contents. Meanwhile, the MAS-added noodles contain less RDS than the wheat noodles, which was observed to decrease by increasing the MAS content. According to Remya et al. [14], chemically modified starch treated with an acid (such as citric acid) forms mono-, di-, and tri-ester bonds through reactions between the hydroxyl groups of the starch and acid functional groups. Subsequent thermal reactions induce further cross-linking, thereby enhancing the RS content. In a similar manner, MAS-added noodles, which contain ester bonds and cross-links, exhibit higher RS contents and lower RDS levels with increasing MAS content.

MAS becomes extensively esterified and cross-linked through intense acid treatment, which ensures structural stability, even during cooking, while also maintaining a high RS content. However, excessive esterification and cross-linking suppress starch-granule swelling, degrade viscosity, and weaken the dough-binding capacity, leading to inferior noodle quality. Therefore, 10% added MAS is considered to be optimal; this level balances noodle quality against low-calorie functionality while delivering a high RS content and a noodle quality that is comparable to that of wheat noodles.

## 4. Conclusions

In this study, we produced noodles using MAS, which is mostly composed of RS (99.5%), owing to its low-calorie functionality. MAS was prepared by physicochemically modifying native wheat starch with 4 M malic acid at 130 °C for 7 h, which improved the thermal stability of the flour. Noodles were prepared by partially substituting wheat flour with 5%, 10%, 20%, or 30% MAS. The effects of MAS on the quality and starch-digestibility of cooked noodles were investigated to determine the optimal formulation. Noodles became increasingly white with increasing MAS content, whereas lower overall lightness, redness, and yellowness were observed following cooking. Extensive MAS esterification and cross-linking lead to a lower dough-binding capacity and a decline in texture, extensibility, and cooking properties compared to those of wheat noodles, as well as more leached solids. However, higher levels of MAS led to cooked noodles with higher RDS and lower RS contents, highlighting its potential as a low-calorie food ingredient for use in the food industry. Based on these findings, wheat flour containing 10% MAS (MAS-10) was identified as being optimal, as noodle quality was maintained while delivering a significantly higher RS content; indeed, the MAS-10 noodles exhibited properties comparable to those of commercially available wheat flour noodles. Future studies aimed at improving the digestibility and physicochemical properties of MAS are expected to contribute to the development of functional MAS materials with health benefits. In addition, expanding research into various food applications is expected to lead to the development of new low-calorie functional food products.

## Figures and Tables

**Table 1:** Formulations of noodles with different MAS contents.

Sample	Ingredients (g)				
	Wheat Flour	Modified Starch	Native Starch	Salt	Water
Wheat	1000	–	–	10.0	340
Control	800	–	200	10.0	340
MAS-5	950	50	–	10.0	340
MAS-10	900	100	–	10.0	340
MAS-20	800	200	–	10.0	340
MAS-30	700	300	–	10.0	340

**Table 2:** Texture analyzer operating condition for cooked MAS-added noodles.

Item	Condition	
Test type	TPA test	Tensile strength test
Measurement type	Two bite compression	Return to start
Sample size	3.0 × 2.2 × 50 mm	3.0 × 2.2 × 300 mm
Probe	35 mm dia, circle	Spaghetti/Noodle tensile rig
Test speed	1.0 mm/s	2.0 mm/s
Deformation	50%	120 mm
Trigger force	0.049 N	0.049 N

**Table 3:** Hunter color values of noodles with different MAS contents.

Sample		Hunter’s Color Value			
		L*	a*	b*	Δ*E*
Uncooked noodle sheet	Wheat	76.5 ± 0.76 ^b^	0.37 ± 0.06 ^b^	16.4 ± 1.12 ^b^	78.2 ± 0.97 ^b^
	Control	73.1 ± 0.54 ^a^	−1.76 ± 0.13 ^a^	16.1 ± 0.16 ^ab^	74.9 ± 0.55 ^a^
	MAS-5	82.1 ± 0.78 ^c^	0.74 ± 0.10 ^c^	22.3 ± 0.44 ^d^	85.1 ± 0.65 ^c^
	MAS-10	85.4 ± 0.52 ^d^	0.52 ± 0.02 ^b^	18.3 ± 0.12 ^c^	87.3 ± 0.53 ^d^
	MAS-20	86.5 ± 0.31 ^e^	0.47 ± 0.02 ^b^	17.0 ± 0.07 ^b^	88.2 ± 0.31 ^d^
	MAS-30	88.2 ± 0.54 ^f^	0.35 ± 0.16 ^b^	15.3 ± 0.34 ^a^	89.5 ± 0.54 ^e^
Cooked noodle sheet	Wheat	69.0 ± 1.29 ^d^	−6.05 ± 0.84 ^b^	9.72 ± 0.83 ^b^	70.0 ± 1.28 ^e^
	Control	54.0 ± 2.00 ^c^	−11.6 ± 0.86 ^a^	3.62 ± 1.02 ^a^	55.4 ± 1.77 ^d^
	MAS-5	66.7 ± 2.08 ^d^	−4.10 ± 0.68 ^c^	19.7 ± 0.68 ^d^	69.7 ± 2.15 ^e^
	MAS-10	26.7 ± 0.50 ^b^	−3.30 ± 0.31 ^cd^	16.1 ± 0.44 ^c^	31.4 ± 0.44 ^c^
	MAS-20	4.58 ± 0.77 ^a^	−2.85 ± 0.49 ^d^	10.2 ± 0.81 ^b^	11.5 ± 0.93 ^b^
	MAS-30	2.44 ± 1.25 ^a^	−1.55 ± 0.05 ^e^	4.55 ± 0.52 ^a^	5.45 ± 0.92 ^a^

^a–f^ The values with different superscripts within a column are significantly different (*p* < 0.05) by Duncan’s multiple range test.

**Table 4:** Textural profiles of cooked noodles with different MAS contents.

Sample	TPA				
	Hardness (N)	Springiness	Cohesiveness	Chewiness (N·mm)	Gumminess (N)
Wheat	28.3 ± 0.88 ^c^	0.116 ± 0.015 ^a^	0.155 ± 0.003 ^d^	0.502 ± 0.084 ^d^	4.48 ± 0.077 ^d^
Control	15.9 ± 2.09 ^b^	0.092 ± 0.007 ^b^	0.125 ± 0.006 ^c^	0.184 ± 0.039 ^bc^	2.00 ± 0.332 ^b^
MAS-5	28.2 ± 1.62 ^c^	0.082 ± 0.002 ^ab^	0.107 ± 0.009 ^b^	0.250 ± 0.022 ^c^	3.03 ± 0.245 ^c^
MAS-10	27.1 ± 2.08 ^c^	0.083 ± 0.007 ^ab^	0.107 ± 0.011 ^b^	0.241 ± 0.033 ^c^	2.88 ± 0.211 ^c^
MAS-20	17.0 ± 1.21 ^b^	0.083 ± 0.011 ^ab^	0.097 ± 0.010 ^ab^	0.138 ± 0.033 ^ab^	1.65 ± 0.191 ^b^
MAS-30	11.3 ± 0.70 ^a^	0.073 ± 0.006 ^a^	0.085 ± 0.008 ^a^	0.070 ± 0.010 ^a^	0.95 ± 0.048 ^a^

^a–d^ The values with different superscripts within a column are significantly different (*p* < 0.05) by Duncan’s multiple range test.

**Table 5:** Tension profiles of cooked noodles with different MAS contents.

Sample	Tension	
	Force (N)	Distance (mm)
Wheat	0.379 ± 0.018 ^e^	46.0 ± 3.56 ^e^
Control	0.106 ± 0.012 ^b^	17.5 ± 1.68 ^b^
MAS-5	0.149 ± 0.006 ^d^	25.2 ± 2.84 ^d^
MAS-10	0.124 ± 0.007 ^c^	20.7 ± 0.35 ^c^
MAS-20	0.063 ± 0.004 ^a^	4.70 ± 3.39 ^a^
MAS-30	N.D.	N.D.

^a–e^ The values with different superscripts within a column are significantly different (*p* < 0.05) by Duncan’s multiple range test.

**Table 6:** Cooking characteristics of noodles with different MAS contents.

Sample	Cooked Noodle			Cooking Water
	Weight (g)	Volume (mL)	Water Absorption (%)	Turbidity (675 nm)
Wheat	54.7 ± 0.02 ^e^	195 ± 1.00 ^d^	118 ± 1.33 ^e^	0.185 ± 0.031 ^a^
Control	66.0 ± 0.03 ^f^	211 ± 0.58 ^e^	164 ± 2.85 ^f^	0.194 ± 0.020 ^a^
MAS-5	48.8 ± 0.01 ^d^	193 ± 0.58 ^d^	94.9 ± 1.32 ^e^	0.262 ± 0.019 ^a^
MAS-10	40.9 ± 0.01 ^c^	188 ± 1.53 ^c^	63.4 ± 3.31 ^d^	0.422 ± 0.044 ^b^
MAS-20	33.9 ± 0.10 ^b^	177 ± 1.00 ^b^	35.0 ± 1.48 ^b^	0.637 ± 0.047 ^c^
MAS-30	28.4 ± 0.02 ^a^	172 ± 1.00 ^a^	13.5 ± 3.56 ^a^	1.282 ± 0.080 ^d^

^a–f^ The values with different superscripts within a column are significantly different (*p* < 0.05) by Duncan’s multiple range test.

**Table 7:** RDS, SDS, and RS contents of cooked noodles with different MAS contents.

Sample	RDS (%)	SDS (%)	RS (%)
Wheat	78.9 ± 0.81 ^f^	2.96 ± 0.54 ^bc^	18.1 ± 0.96 ^a^
Control	81.5 ± 0.96 ^e^	0.57 ± 0.69 ^a^	17.9 ± 0.64 ^a^
MAS-5	75.5 ± 0.27 ^d^	2.57 ± 0.30 ^b^	22.0 ± 0.25 ^b^
MAS-10	71.0 ± 0.65 ^c^	3.08 ± 0.49 ^bc^	25.9 ± 0.54 ^c^
MAS-20	61.6 ± 1.01 ^b^	3.73 ± 0.52 ^c^	34.7 ± 0.13 ^d^
MAS-30	50.9 ± 0.44 ^a^	5.74 ± 0.24 ^d^	43.3 ± 0.67 ^e^

^a–f^ The values with different superscripts within a column are significantly different (*p* < 0.05) by Duncan’s multiple range test.

## Data Availability

The original contributions presented in this study are included in the article. Further inquiries can be directed to the corresponding author.

## Abbreviations

The following abbreviations are used in this manuscript:

MAS	malic acid-modified starch
RDS	rapidly digestible starch
SDS	slowly digestible starch
RS	resistant starch

## References

[1] Bertoft E. (2017). Understanding starch structure: Recent progress. Agronomy.

[2] Copeland L., Blazek J., Salman H., Tang M.C. (2009). Form and functionality of starch. Food Hydrocoll..

[3] Wang S., Guo P., Wang S. (2020). Botanical sources of starch. Starch Structure, Functionality and Application in Foods.

[4] Aller E.E., Abete I., Astrup A., Martinez J.A., van Baak M.A. (2011). Starches, sugars and obesity. Nutrients.

[5] Seung D. (2020). Amylose in starch: Towards an understanding of biosynthesis, structure and function. New Phytol..

[6] Svihus B., Hervik A.K. (2016). Digestion and metabolic fates of starch, and its relation to major nutrition-related health problems: A review. Starch-Stärke.

[7] Nag S., Majumder S. (2023). Starch, gallic acid, their inclusion complex and their effects in diabetes and other diseases—A review. Food Sci. Nutr..

[8] Dundar A.N., Gocmen D. (2013). Effects of autoclaving temperature and storing time on resistant starch formation and its functional and physicochemical properties. Carbohydr. Polym..

[9] Na J.H., Jeong G.A., Park H.J., Lee C.J. (2021). Impact of esterification with malic acid on the structural characteristics and in vitro digestibilities of different starches. Int. J. Biol. Macromol..

[10] Vatanasuchart N., Niyomwit B., Wongkrajang K. (2012). Resistant starch content, in vitro starch digestibility and physico-chemical properties of flour and starch from Thai bananas. Maejo Int. J. Sci. Technol..

[11] Birt D.F., Boylston T., Hendrich S., Jane J.-L., Hollis J., Li L., McClelland J., Moore S., Phillips G.J., Rowling M. (2013). Resistant starch: Promise for improving human health. Adv. Nutr..

[12] Wolf B.W., Wolever T.M.S., Bolognesi C., Zinker B.A., Garleb K.A. (2001). Glycemic response to a rapidly digested starch is not affected by the addition of an indigestible dextrin in humans. Nutr. Res..

[13] Xie X., Liu Q. (2004). Development and physicochemical characterization of new resistant citrate starch from different corn starches. Starch-Stärke.

[14] Remya R., Jyothi A.N., Sreekumar J. (2018). Effect of chemical modification with citric acid on the physicochemical properties and resistant starch formation in different starches. Carbohydr. Polym..

[15] Klostermann C., Buwalda P., Leemhuis H., de Vos P., Schols H., Bitter J. (2021). Digestibility of resistant starch type 3 is affected by crystal type, molecular weight and molecular weight distribution. Carbohydr. Polym..

[16] Gutiérrez T.J., Tovar J. (2021). Update of the concept of type 5 resistant starch (RS5): Self-assembled starch V-type complexes. Trends Food Sci. Technol..

[17] Khawas P., Deka S.C. (2017). Effect of modified resistant starch of culinary banana on physicochemical, functional, morphological, diffraction, and thermal properties. Int. J. Food Prop..

[18] Perera A., Meda V., Tyler R. (2010). Resistant starch: A review of analytical protocols for determining resistant starch and of factors affecting the resistant starch content of foods. Food Res. Int..

[19] Hung P.V., Vien N.L., Lan Phi N.T. (2016). Resistant starch improvement of rice starches under a combination of acid and heat-moisture treatments. Food Chem..

[20] Lockyer S., Nugent A. (2017). Health effects of resistant starch. Nutr. Bull..

[21] Giuberti G., Marti A., Fortunati P., Gallo A. (2017). Gluten free rice cookies with resistant starch ingredients from modified waxy rice starches: Nutritional aspects and textural characteristics. J. Cereal Sci..

[22] Pang Z., Xu R., Luo T., Che X., Bansal N., Liu X. (2019). Physiochemical properties of modified starch under yogurt manufacturing conditions and its relation to the properties of yogurt. J. Food Eng..

[23] Majzoobi M., Hedayati S., Habibi M., Ghiasi F., Farahnaky A. (2014). Effects of corn resistant starch on the physicochemical properties of cake. J. Agric. Sci. Technol..

[24] Yousif E., Gadallah M.E., Sorour A.M. (2012). Physico-chemical and rheological properties of modified corn starches and its effect on noodle quality. Ann. Agric. Sci..

[25] Chae R., Jeong G.A., Kim H.-J., Lee C.J. (2023). Quality characteristics of cookies added with octenyl succinyl anhydride-modified wheat starch. Food Eng. Prog..

[26] Alexander V., Sobhi B., Joseph S., Beta T., Malunga L.N. (2025). Exploring the noodle-making potential and digestibility of native oat starch and citric acid cross-linked resistant oat starch. Cereal Chem..

[27] Kim H.R., Jeong G.A., Bae J.-E., Hong J.S., Choi H.-D., Lee C.J. (2022). Impact of chemical modification by immersion with malic acid on the physicochemical properties and resistant starch formation in rice. J. Food Sci..

[28] Lee C.J., Na J.H., Park J.-Y., Chang P.-S. (2019). Structural Characteristics and In Vitro Digestibility of Malic Acid-Treated Corn Starch with Different pH Conditions. Molecules.

[29] Mansur A.R., Jeong G.A., Lee C.J. (2022). Preparation, physicochemical properties, and in vivo digestibility of thermostable resistant starch from malic acid-treated wheat starch. Food Res. Int..

[30] Kim H.-S., Huber K.C. (2008). Channels within soft wheat starch A-and B-type granules. J. Cereal Sci..

[31] Kim S.-K., Kim H.-R., Bang J.-B. (1996). Effects of alkaline reagent on the rheological properties of wheat flour and noodle property. Korean J. Food Sci. Technol..

[32] Englyst H.N., Kingman S., Cummings J. (1992). Classification and measurement of nutritionally important starch fractions. Eur. J. Clin. Nutr..

[33] Shin S.I., Lee C.J., Kim D.-I., Lee H.A., Cheong J.-J., Chung K.M., Baik M.-Y., Park C.S., Kim C.H., Moon T.W. (2007). Formation, characterization, and glucose response in mice to rice starch with low digestibility produced by citric acid treatment. J. Cereal Sci..

[34] Yaver E., Bilgiçli N. (2021). Effect of ultrasonicated lupin flour and resistant starch (type 4) on the physical and chemical properties of pasta. Food Chem..

[35] Jeong G., Chae R., Lee C. (2023). Digestibility and quality characteristics of noodles added with octenyl succinic anhydride-modified wheat starch. Food Eng. Prog..

[36] Lin D., Zhou W., Yang Z., Zhong Y., Xing B., Wu Z., Chen H., Wu D., Zhang Q., Qin W. (2019). Study on physicochemical properties, digestive properties and application of acetylated starch in noodles. Int. J. Biol. Macromol..

[37] Liu J., Meenu M., Xu B. (2020). Effect of unripe banana flour and wheat gluten on physicochemical characteristics and sensory properties of white salted noodles. J. Food Process. Preserv..

[38] Li M., Sun Q.-J., Han C.-W., Chen H.-H., Tang W.-T. (2018). Comparative study of the quality characteristics of fresh noodles with regular salt and alkali and the underlying mechanisms. Food Chem..

[39] Lee C.H., Cho J.K., Lee S.J., Koh W., Park W., Kim C.H. (2002). Enhancing β-carotene content in Asian noodles by adding pumpkin powder. Cereal Chem..

[40] Hong J., Li C., An D., Liu C., Li L., Han Z., Zeng X.A., Zheng X., Cai M. (2020). Differences in the rheological properties of esterified total, A-type, and B-type wheat starches and their effects on the quality of noodles. J. Food Process. Preserv..

[41] Hoseney R.C. (1994). Principles of Cereal Science and Technology.

[42] Obadi M., Xu B. (2021). Review on the physicochemical properties, modifications, and applications of starches and its common modified forms used in noodle products. Food Hydrocoll..

[43] Jeong G.A., Han S.H., Park J.Y., Shin Y.L., Lee S.J., Lee C.J. (2019). Quality characteristics of noodles supplemented with rice flour and alkaline reagent. Korean J. Food Sci. Technol..

[44] Shukri R., Shi Y.C. (2017). Structure and pasting properties of alkaline-treated phosphorylated cross-linked waxy maize starches. Food Chem..

